# A *Mycobacterium tuberculosis* Sigma Factor Network Responds to Cell-Envelope Damage by the Promising Anti-Mycobacterial Thioridazine

**DOI:** 10.1371/journal.pone.0010069

**Published:** 2010-04-08

**Authors:** Noton K. Dutta, Smriti Mehra, Deepak Kaushal

**Affiliations:** 1 Division of Bacteriology and Parasitology, Tulane National Primate Research Center, Covington, Louisiana, United States of America; 2 DNA Microarray and Expression Core, Tulane National Primate Research Center, Covington, Louisiana, United States of America; 3 Department of Microbiology and Immunology, Tulane University School of Medicine, New Orleans, Louisiana, United States of America; University of California Merced, United States of America

## Abstract

**Background:**

Novel therapeutics are urgently needed to control tuberculosis (TB). Thioridazine (THZ) is a candidate for the therapy of multidrug and extensively drug-resistant TB.

**Methodology/Principal Findings:**

We studied the impact of THZ on *Mycobacterium tuberculosis* (*Mtb*) by analyzing gene expression profiles after treatment at the minimal inhibitory (1x MIC) or highly inhibitory (4x MIC) concentrations between 1–6 hours. THZ modulated the expression of genes encoding membrane proteins, efflux pumps, oxido-reductases and enzymes involved in fatty acid metabolism and aerobic respiration. The Rv3160c-Rv3161c operon, a multi-drug transporter and the Rv3614c/3615c/3616c regulon, were highly induced in response to THZ. A significantly high number of *Mtb* genes co-expressed with σ^B^ (the σ^B^ regulon) was turned on by THZ treatment. σ^B^ has recently been shown to protect *Mtb* from envelope-damage. We hypothesized that THZ damages the *Mtb* cell-envelope, turning on the expression of the σ^B^ regulon. Consistent with this hypothesis, we present electron-microscopy data which shows that THZ modulates cell-envelope integrity. Moreover, the *Mtb* mutants in σ^H^ and σ^E^, two alternate stress response sigma factors that induce the expression of σ^B^, exhibited higher sensitivity to THZ, indicating that the presence and expression of σ^B^ allows *Mtb* to resist the impact of THZ. Conditional induction of σ^B^ levels increased the survival of *Mtb* in the presence of THZ.

**Conclusions/Significance:**

THZ targets different pathways and can thus be used as a multi-target inhibitor itself as well as provide strategies for multi-target drug development for combination chemotherapy. Our results show that the *Mtb* sigma factor network comprising of σ^H^, σ^E^ and σ^B^ plays a crucial role in protecting the pathogen against cell-envelope damage.

## Introduction

Tuberculosis (TB) is caused by *Mtb*, one of the most successful pathogens of humanity. It leads to the death of about 1.8 million humans annually. The emergence of multidrug resistant (MDR) strains of *Mtb*, synergy between TB and AIDS and the lack of effective vaccines and chemotherapeutics has exacerbated the situation [1]. A major problem for the control of TB is the requirement of drug regimens for six to nine months. These lengthy regimens lead to non-compliance with therapy, relapse and development of drug-resistance. In order to shorten the duration of therapy, novel drugs that are active against *Mtb*, which act through mechanisms different from those employed by the existing frontline and secondary anti-TB drugs are urgently needed. The “non-antibiotics” class of compounds [2], which act by either enhancing antibiotic activity [3]–[5] or reversal of antibiotic resistance [6]–[9], or by induction and control of efflux pumps [10], have the potential to act as the next generation of anti-TB drugs. In fact, the use of the anti-psychotic phenothiazine, thioridazine (THZ) for therapy of multidrug and extensively drug-resistant tuberculosis infections is now being seriously considered [11]–[13]. THZ has broad-spectrum antibacterial activity against *Mtb* [5], [13]. It appears to be equally active on starved *Mtb*, which represents the persistent state of the pathogen, as it is during log phase growth. This is not the case for frontline anti-TB drugs rifampin (RIF) or isoniazid (INH), which have little or no activity respectively, on starved cells [14]. Synergistic activity at the MIC level between rifampicin (RIF) and streptomycin (STR), but not INH, and the phenothiazines has been reported [5]. MICs for phenothiazines are much higher than the corresponding values in macrophages, since THZ concentrates inside these host cells [15]. The MICs in macrophages for inhibiting *Mtb* growth have been reported as 0.23–3.6 mg/ml [10] and 0.1 mg/ml [15]. Equally significant is the fact that at these concentrations, there were no cytotoxic effects on the macrophages [15]. Finally, Bate *et al*. [16] demonstrated that novel phenothiazine derivatives inhibited *Mtb* in the non-replicating state at MICs that were lower than those under actively growing conditions as a ‘macrophage modulator’ [15], [17], [18]. THZ significantly reduced the number of colony forming units (CFU) retrieved from the lungs of mice infected with *Mtb* (10^6^ CFU/ml, i.p.) within one month at a daily dose of 0.5 mg/day [19]. Phenothiazines in general and THZ in particular, exert their anti-TB effects via calmodulin [20], [21] or by inhibiting NADH2-menaquinoneoxidoreductase (Ndh2) [22]–[24].

*Mtb* responds to changes in its environment by altering the level of expression of critical genes that transduce such signals into metabolic changes favoring continued growth and survival. Sigma (σ) factors are a class of transcription factors which control bacterial gene-expression [25]. Typically most eubacteria encode a principal sigma factor and a variable number of alternate sigma factors, which control responses to specific environmental stimuli and adaptation to stress [26]. The temporal expression of specific regulons controlled by one or more alternate sigma factors likely allows *Mtb* to survive in multiple phases of TB [27]. *Mtb* sigma factors σ^H^, σ^E^ and σ^B^ are well studied. The expression of the latter can be triggered by either σ^H^ or σ^E^ [28]–[30]. Thus, there exists a network of these three factors- σ^H^, σ^E^ and σ^B^, with overlapping functions. σ^H^ is induced after heat, redox, nitrosative and acid stress [28], [29], [31], and phagocytosis [32], [33]. σ^H^ directly regulates the transcription of 31 genes, including theσ^E^, σ^B^, *gro*EL/ES and the thioredoxin regulons [28]. σ^E^ is also induced upon uptake by macrophages [32], and upon treatment with hydrogen-peroxide [34], and SDS [35]. σ^E^ regulates the expression of at least 23 genes, including σ^B^, *hsp* and *htp*X. σ^E^ is transcribed in either a σ^H^ [28], [29] or an MprAB-dependent [36] manner. σ^B^ is the minor principal sigma factor of *Mtb* and appears to play a key role in defense against cell-envelope damage [37]. The expression of these sigma factors is also altered by treatment with various anti-bacterial compounds [38], indicating that this sigma-factor network plays a role in defense against these anti-*Mtb* mechanisms.

System-wide screens can help predict specific pathways and targets that individual anti-bacterial compounds interact with. In turn, this has the potential to identify newer druggable target genes, pathways and networks. Identification of clusters of coordinately regulated genes for drug mechanism of action is a rational approach to the selection of critical drug targets. We conducted an in-depth investigation on the mechanism of action of THZ by exploring drug specific changes. Some of these changes may reflect the mode of action of THZ. Treatment with THZ caused damage to the *Mtb* cell-envelope and altered its energy metabolism. Our results show that the *Mtb* σ^H^/σ^E^/σ^B^ network is perturbed by treatment with THZ, and plays an important role in the survival of the pathogen under such adverse conditions.

## Methods

### Bacterial culture and drug treatment

THZ (Sigma), dissolved in water, at a final concentration of 1xMIC (10 µg/ml) or 4xMIC (40 µg/ml) was added to *Mtb* (CDC1551) cultures grown in Middlebrook 7H9-albumin-dextrose-catalase-Tween 80-glycerol broth at an OD_546_ of 0.5 - 0.6 and the cultures were aerated by shaking at 37°C for additional 1–6 h. Control (untreated) samples were treated with the appropriate solvent vehicle.

### RNA extraction, cDNA labeling and Microarrays

In order to profile transcriptome-wide changes in response to THZ treatment, *Mtb* cultures were actively cultured to log-phase, prior to addition of the drug. RNA was isolated using the Trizol-beadbeater method [30]. *Mtb* whole genome DNA microarrays comprised of 70mer probes representing 4,269 *Mtb* (H37Rv and CDC1551) genes, plus 26 controls were obtained from “TB Vaccine Testing and Research Materials” Contract, Colorado State University. Experiments were performed according to the standard protocols disseminated by the Mycobacteria Research Lab at the Colorado State University (http://www.cvmbs.colostate.edu/mip/tb/pdf/arrays.pdf). Data was analyzed using S^+^ scripts within Spotfire DecisionSite for Microarray Analysis (TIBCO-Spotfire) to determine significant expression changes, adjusting for intensity bias using Locally Weighted Scatter-plot Smoothing (LOWESS) normalization [30]. Genes whose expression was altered in magnitude by at least 75% in each of the two biological replicate arrays, relative to untreated control in a statistically significant manner (one way analysis of variance, *P*≤0.05) were included in our analysis.

### Public access to microarray data

Our microarray experiments and data analysis were conducted strictly in a MIAME compliant manner, per the suggestions of the MGED society (http://www.mged.org/Workgroups/MIAME/miame.html). Our raw and normalized microarray data is publically available at the Gene Expression Omnibus (GEO) database (accession number GSE16626).

### Verification of microarray data by quantitative real-time RT-PCR

RT-PCR (Applied Biosystems ABI 7700) was performed with cDNA corresponding to 50 ng of each independent RNA sample, using the SYBR green Supermix kit (Applied Biosystems) with specific primers for each target (Table S1). Relative expression levels were normalized using σ^A^ as an invariant transcript, and data was analyzed using the ΔΔCt method as previously described [30]. The average relative expression levels and the standard deviations were determined for three independent experiments.

### Analysis of the effect of THZ on *Mtb* cell-envelope by electron microscopy

Log-phase cultures of unexposed *Mtb* CDC 1551 (untreated control) and following incubation for 6 hr and 24 hr with 1x MIC/4x MIC of THZ, respectively were fixed overnight in 1.25% glutaraldehyde-2% formaldehyde in 0.1 M sodium cacodylate, pH 7.3. They were washed in 2 changes of 0.1 M sodium cacodylate, pH 7.3, passed through an ethanol dehydration process, infiltrated with Epon Araldite resin, and polymerized overnight at 70°C. Sections were cut on a MTXL ultratome (RMC Products), placed on 150-mesh copper grids, stained with 5% uranyl acetate and lead citrate, and examined with a Zeiss 10 transmission electron microscope [39].

### Comparison of the viability of *Mtb*, its isogenic Δ–σ^H^ and Δ–σ^E^ mutants and the strain conditionally over-expressing σ^B^, in the presence of THZ

A portion of recently frozen stocks of *Mtb* CDC1551 [40], the isogenic *Mtb* CDC1551 Δ–σ^H^ mutant, the isogenic *Mtb* CDC1551 Δ–σ^E^ mutant and the *Mtb* CDC1551 σ^B^ Knock-In (KI) strain [41] were inoculated into 5 ml of Middlebrook 7H9 broth (7H9) supplemented with 10% albumin dextrose catalase, v/v, and 0.05% Tween-80, v/v (Sigma), and incubated at 37°C for 5 days. The induction of σ^B^ in the σ^B^ (KI) strain was performed as described [41]. Briefly, the culture was grown to mid-log phase, and acetamide was added to a final concentration of 0.2%. The culture was then transferred into 50 ml of 7H9 media and incubated at 37°C with 50 rpm shaking until growth reached log phase. *Mtb* CDC1551 is susceptible to THZ (MIC: 10 µg/ml, MBC: 40 µg/ml). THZ was added to the culture at final concentrations of 0.1x, 1xMIC, 4xMIC, and the cultures were then incubated at 37°C up to 7 days. Control (untreated) samples were treated with the appropriate solvent vehicle.

### NADH/NAD^+^ measurement

This assay was adapted from Boshoff et al. [22]. Briefly, *Mtb* CDC1551 and σ^B^ over-expressing (KI) cultures were grown to an *A*_650_ of 0.3 and treated with THZ (10 µg/ml) for 3 h. Cells were rapidly harvested and pellets were suspended in either acid (0.2 M HCl) or base (0.2 M NaOH) for NAD or NADH, respectively. Nucleotide extraction and nucleotide cycling assay were performed as previously described [42]. Data represent the average of 3 independent experiments.

## Results

### Global transcriptional response of *Mtb* to THZ treatment

The *Mtb* CDC1551 strain [43] is sensitive [44] to THZ (MIC: 10 µg/ml) [5], [13]. The drug showed noteworthy inhibitory action (MIC_90_ = 10 µg/ml) against this strain, demonstrated cidal (minimum bactericidal concentration  = 40 µg/ml) activity. While the transcriptional response of *Mtb* to a concentration15 µg/ml of THZ has been reported [23], we performed an in-depth study of the entire *Mtb* transcriptome to two concentrations, 1x MIC (basal level) and 4x MIC (high level) of THZ during a number of early time points (0–6 h post-treatment). The time points were chosen based on published data [5], [18], [22], [45]. Our experiment was designed to ensure that the primary effects of the drugs and any dose responses would be captured.

Treatment with THZ had an immediate and profound effect on the global transcription in *Mtb* (Table S2 and Figure S1). Venn-diagrams show an overlap in the *Mtb* gene-expression program at different time-points (Figure S2). A total of 10 genes (Rv1057, Rv1497, Rv2483c, Rv2710 (σ^B^), Rv2711, Rv2744c, Rv2745c, Rv3065 (*emr*E), Rv3160c and Rv3161c) were commonly induced at all time-points upon 1x THZ treatment (Table 1). The expression of a total of 19 genes (Rv0251c, Rv1040c, Rv1057, Rv1403c, Rv1404, Rv1461, Rv1463, Rv1466, Rv1497, Rv1854c, Rv2053c, Rv2694c, Rv2710 (σ^B^), Rv2743c, Rv2745c, Rv3065 (*emr*E), Rv3160c, Rv3161c and Rv3162c) was induced after exposure of THZ at 4XMIC. Thus, Rv1057, Rv1497, Rv2710 (σ^B^), Rv2745c, Rv3065, Rv3160c and Rv3161c were induced at all time-points and in response to both 1x and 4x THZ (Table 1).

**Table 1:** Genes induced in all condition tested.

Condition	Rv number	Gene^*^	Description	Functional Category	Fold induction (p value) [qRT-PCR]			
					1 h	2 h	4 h	6 h
IXMIC up regulation	Rv1057		Conserved hypothetical	10	3.69(0.01)	9.79(0.004)	9.4(0.005)	1(0.003)
	Rv1497	***lip*** **L**	Probable esterase LIPL	3	2.154(0.01)	3.21(0.01)	6.4(0.002)	4(0.008)
	Rv2483c	*pls*C	Possible transmembrane phospholipid	1	2.292(0.01)	1.94(0.04)	3.3(0.0021)	4.3(0.01)
	Rv2710	***sig*** **B**	Sigma Factor B	2	2.449(0.01)[2.11]	3.98(0.004)[9.27]	5(0.01) [1.02]	7(0.01) [1.47]
	Rv2711	*ide*R	Iron-dependent repressor and activator ider	9	1.832(0.02)	1.95(0.03)	2.3(0.009)	2.9(0.006)
	Rv2744c		Conserved 35 KDA alanine rich protein	10	2.17(0.03)	2.26(0.03)	9.1(0.003)	5.4(0.007)
	Rv2745c		Possible transcriptional regulatory protein	9	2.02(0.04) [1.31]	3.32(0.01) [5.24]	35(0.001) [3.27]	8(0.005) [19.09]
	Rv3065	***emr*** **E**	Multidrug-transport integral membrane protein MMR	3	10.81(0.0009) [4.86]	1.88(0.001) [2.27]	23(0.0004)[41.22]	31(0.0002)[6.04]
	Rv3160c		Transcriptional regulatory protein	9	10.95(0.0039) [11.26]	8.39(0.006)	19(0.0006)	39(0.001)
	Rv3161c		Possible dioxygenase	7	76.8(4.70E-05) [41.65]	15.6(0.008)	38(1.85E-05)	74(0.0005)
4XMIC up regulation	Rv0251c	*hsp*	Hypothetical exported protein	3	10.82(0.0005) [2.93]	17(0.0009) [3.58]	29(0.0003) [5.90]	36(0.04) [7.53]
	Rv1040c		PE family protein	6	1.983(0.04)	4.49(2.44E-06)	2.4(0.007)	2.3(0.0004)
	Rv1057		Conserved hypothetical protein	10	9.5(0.003)	12.7(0.002)	8.3(0.01)	7.9(0.003)
	Rv1403c		Putative methyltransferase	7	1.73(0.04)	6.41(0.002)	7(0.0004)	3.8(0.002)
	Rv1404		Probable transcriptional regulatory protein	9	2.53(0.02)	2.82(0.01)	3.7(0.01)	3.1(0.01)
	Rv1461		Conserved hypothetical protein	10	2.98(0.01)	4.98(0.01)	7.8(0.002)	5.6(0.003)
	Rv1463		Probable conserved ATP-binding protein	3	2.92(0.01653)	4.83(0.004)	5.4(0.01)	3.9(0.01)
4XMIC up regulation	Rv1466		Conserved hypothetical protein	10	2.97(0.04)	3.91(0.01)	5.7(0.01)	4.2(0.01)
	Rv1497	***lip*** **L**	Probable esterase LIPL	3	5.02(0.01)	5.59(0.007)	2.8(0.01)	2.6(0.01)
	Rv1854c	*ndh*	Probable NADH dehydrogenase NDH	7	3.94(0.004)	2.12(0.01)	3.3(0.01)	2.1(0.01)
	Rv2053c		Probable transmembrane protein	3	10.19(0.001)	4.32(0.01)	2(0.0008)	2.8(0.04)
	Rv2694c		Conserved hypothetical protein	10	3.04(0.01)	4.69(0.009)	3.1(0.02)	3.2(0.004)
	Rv2710	***sig*** **B**	RNA polymerase sigma factor SIGB	2	10.34(0.004) [17.61]	14.2(0.01) [12.32]	9.5(0.0006) [10.73]	5.7(0.0009) [12.60]
	Rv2743c		possible conserved transmembrane alanine rich protein	3	3.74(0.01)	3.3(0.01)	3(0.02)	4(0.03)
	Rv2745c		Possible transcriptional regulatory protein	9	5.16(0.01) [3.43]	5.83(0.02) [11.33]	146(0.001) [13.27]	10(0.005) [89.55]
	Rv3065	***emr*** **E**	Multidrugs-transport integral membrane protein MMR	3	24.66(0.001) [15.44]	24(0.0006) [5.20]	32(0.01) [26.40]	59(0.003)[100.60]
	Rv3160c		Possible transcriptional regulatory protein	9	66.98(0.001) [26.56]	31.4(0.0005)	23(0.0008)	21(0.0005)
	Rv3161c		Possible dioxygenase	7	237.3(0.01) [155.54]	28.8(0.02)	48(0.0003)	56(0.003)
	Rv3162c		Possible integral membrane protein	3	2 (0.04)	4.3(0.009)	2.9(0.04)	4(0.01)
4XMIC down regulation	Rv2950c	*fadD*29	Probable FATTY-ACID-CoA ligase FADD29	1	−4.39(0.01)	−12.33(0.001)	−3.92(0.008)	−2.30(0.02)
	Rv0170	*mce*D	MCE-family protein MCE1B	0	−7.73(0.003)	−3.15(0.001)	−2.05(0.005)	−2.39(0.003)
	Rv2450c	*rpf*E	Probable resuscitation-promoting factor RPFE	3	−1.87(0.001)	−7.19(0.0001)	−3.76(0.001)	−3.55(5.05E-05)
	Rv3920c		Hypothetical protein similar to JAG protein	10	−1.69(0.009)	−2.77(0.003)	−2.94(0.01)	−3.07(0.002)

Genes are annotated as described by the Pasteur Institute on TubercuList (http://genolist.pasteur.fr/TubercuList).

Enclosed genes found to be induced under both Ix and 4x MIC conditions are in bold.

Values in square brackets represent the fold induction obtained by qRT-PCR.

### Impact of THZ on the transcription of specific *Mtb* networks

The expression of the predicted Rv3160c/Rv3161c operon was induced at all time-points, and at both concentrations, following THZ treatment. The former encodes a TetR family member transcription factor, while the latter encodes for a dioxygenase. This operon is also induced in response to the anti-mycobacterials Triclosan [22], [46] and SRI967 and SRI9190 [47].

Treatment with THZ also caused a profound increase in the expression of the Rv3065 encoded *Emr*E (*Mmr*) multidrugs transport membrane protein. While the expression of only the *emr*E gene was induced at the 1 hr (both 1x and 4x concentrations), the expression of an entire genomic locus containing this gene, Rv3060c-Rv3064c; Rv3065-Rv3067 was induced at 4 and 6 hr time-points. This region includes a putative DeoR family transcription factor encoded by the Rv3066 gene, and a gene belonging to the carbon-starvation induced protein family, encoded by then Rv3063 gene. Another key gene that was induced following THZ treatment at the 1 hr time point is *kgt*P, which encodes the major facilitator super-family protein. Paralogs of this protein are involved in nitrate uptake and nitrite efflux.

At later time-points, we observed the induction of *whi* family members and *kas* operon, also known to be induced in a σ^B^-dependent manner. At 4 and 6 hrs post-THZ treatment we observed a significant induction of ESAT-6 and the Rv3614c-Rv3615c-Rv3616c operon, which forms the Esx-1 secretion apparatus critical for the virulence of *Mtb* via both the export of Esx virulence substrates as well as a major source of potent immunostimulatory antigens [48]. The expression of Rv1057 and *lip*L genes was also induced upon treatment with THZ.

Phenothiazines are potent inhibitors of the type II NADH-ubiquinone dehydrogenase, as well as the membrane-bound succinate dehydrogenase [23]. THZ blocks NADH-dependent O_2_ consumption by *Mtb* membrane [22]–[24], thereby affecting respiratory and other intermediary metabolic activities of the cell. Consistent with this, we observed the over-expression of *ndh* at all time points of 4xMIC treatment. This corroborates the work of Boshoff et al [22]. The expression of several other genes involved in energy metabolism, e.g. NADH dehydrogenases (*nuo*E, *nuo*F and *nuo*G), isocitrate dehydrogenase (*icd*1), NAD-dependent malate reductase (*mez*) and NADPH:quinone oxidoreductase (Rv0149) was also induced by THZ.

The expression of fewer genes was repressed in *Mtb* following THZ treatment (Table 1). Overall, four genes (*fad*D22, *mce*4, *rpf*E and Rv3920c) exhibited a lower level of expression at all four time-points (1–6 hr) following 4x MIC treatment with THZ. The expression of a related gene, also encoding another resuscitation promotion factor, *rpf*C, was repressed upon treatment with 1x MIC THZ, although given the extensive sequence similarity between the *rpf* gene family members, cross-hybridization during microarray experiments can't be ruled out.

### Functional categorization of the post-THZ treatment transcriptome changes in *Mtb*

**Figure 1:**
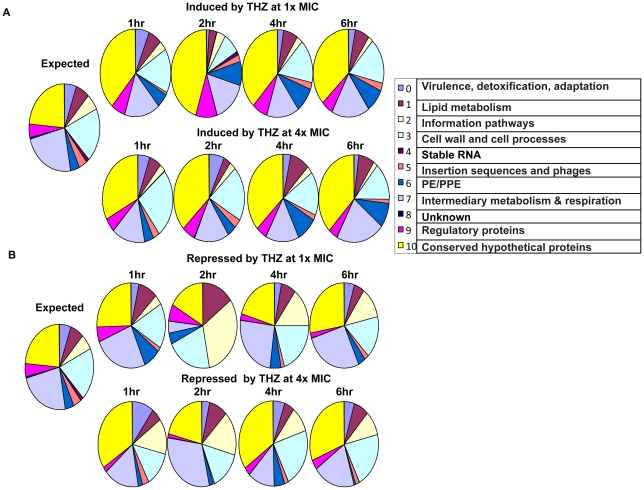
Functional categorization of the global induction (A) or repression (B) in *Mtb* gene-expression following 1x (top panel in both A and B) or 4x THZ (bottom panel in both A and B) treatment is shown as a series of pie charts. The first (leftmost) pie corresponds to an expected distribution of the various functional categories into which the *Mtb* genes are represented based on the total number of genes corresponding to each category. The number of genes from each functional category, whose expression was perturbed in individual THZ treatment experiments, is represented in the subsequent pies. The first functional category (0 = Virulence and detoxification) begins at 12 O Clock in very pie and the subsequent categories are represented in a clock-wise direction.

We analyzed the effect of THZ on *Mtb* gene-expression based on the basis of the various functional categories, to which the various *Mtb* genes belong. The actual number of genes from each functional category that were perturbed was compared to the expected number for the data of a finite size. The expression of fewer than expected genes belonging to the functional category “Intermediary metabolism” and a larger than expected number of genes belonging to the categories “Transcriptional regulators” and “Conserved hypothetical proteins” was induced by THZ treatment (Figure 1A). Differences were also observed in a concentration-dependent manner. Mining of the transcriptome data in the context of functional categories yielded particularly interesting data when applied to genes whose expression was repressed in *Mtb* following THZ treatment (Figure 1B). The expression of a much larger than anticipated number of genes belonging to the “Information pathways” and “Lipid metabolism” categories were repressed at 2 hr post-THZ treatment. These results indicate that in response to THZ treatment, a significant lowering of the expression of genes belonging to many key biosynthetic pathways is observed. Together, these results indicate that THZ treatment causes an immediate spike in the expression of specific transcriptional regulators that mediate the pathogen's response to cell-membrane damage, coupled with a silencing in expensive energetic processes such as lipid-metabolism, and DNA, RNA and protein synthesis. One such transcription factor is σ^B^, which has recently been shown to protect *Mtb* against membrane damaging stress [37], and whose expression was induced in response to all treatments with THZ. Therefore, we decided to study the role played by this transcription factor, in defense against THZ-stress, in detail.

### Impact of THZ on the σ^B^ regulon

A large gene-expression network that was immediately induced following THZ treatment is controlled by the sigma factor σ^B^, which is known to interact in a complex manner with other alternate, extracytoplasmic signal induced sigma factors and transcriptional activators/repressors [28], [29], [41], [49]. Apart from σ^B^ (Rv2710), we also observed the induction of *ide*R (Rv2711), the iron-dependent repressor of siderophore and mycobactin biosynthesis, and *hsp* which codes for a heat shock protein. These genes have previously been shown to be dependent on σ^B^ for their induction [41]. The *htp*X and *clp*B genes were also co-expressed with σ^B^. These genes have previously been observed to be co-expressed with σ^B^ in other experiments where key alternate *Mtb* sigma factors σ^H^ or σ^E^ were induced [28], [30]. The expression of Rv2745c and its adjoining genes (Rv2744c and Rv2743c) was also significantly induced at almost all time-points. Rv2745c is known to be induced in σ^H^ and σ^E^ dependent manner following disulphide or membrane damage stress [30], [37].

Since a large number of the σ^B^ dependent genes appeared to be induced in *Mtb* upon THZ treatment, we decided to analyze the expression of the entire σ^B^ regulon. Using either the Tuberculosis Database (TBDB) or the NIAID BioHealthBase BRC online tool (http://www.biohealthbase.org), we identified 100 *Mtb* genes that have been classified as members of the σ^B^ regulon and which have an extremely high degree of co-expression correlation (0.78601 to 0.40569) with σ^B^. As shown in Table S3A, 89 out of these 100 σ^B^ co-expressed genes were induced by THZ treatment of *Mtb*, at any of the several time points in our experiment, while only 11 out of 100 members of the “σ^B^ regulon” were not induced by THZ. This clearly indicates that the entire σ^B^ regulon is turned on by THZ treatment. Recently, Fontan et al [37] performed an in depth study into the function of σ^B^ in stress response. They studied σ^B^ regulated genes separately during stress with SDS, which induces the expression of σ^B^ via σ^E^ [29], and during stress with diamide, which induces the expression of σ^B^ via σ^H^ [30]. We compared our transcriptome data to that of Fontan et al [37]. As shown in Table S3B, 57 members of the “σ^B^ regulon” out of 73 that were induced in *Mtb* relative to the Δ–σ^B^ mutant by SDS were also induced by THZ treatment. Similarly, 30 members of the “σ^B^ regulon” out of 41 that were induced in *Mtb* relative to the Δ–σ^B^ mutant by SDS were also induced by THZ treatment (Table S3C). These results clearly show that the *Mtb* σ^B^ regulon, which is activated by treatment with SDS or diamide, via either σ^E^ or σ^H^, is also activated by treatment with THZ.

We then compared the transcriptome of *Mtb* treated with THZ to that of *Mtb* treated with vancomycin (VAN) [38], and found a very high degree of overlap between the two treatments. For this purpose, data was obtained from Gene Expression Omnibus (GEO) from the following replicate microarray experiments: 10 x MIC VAN treated vs. untreated *Mtb*, at both 1 and 4 hrs. Raw data was then filtered and analyzed in a manner comparable to that described for our own data. We found that the expression of 160 genes was significantly induced in *Mtb* following VAN treatment at both time points. 37 out of the 100 strongly co-expressed members of the σ^B^ regulon were present in this cluster of 160 genes. Interestingly, the expression of 32 out of these 37 genes was also induced by THZ (Table S3D). These results show that both VAN and THZ exert similar effects on *Mtb*.

### Quantitative PCR based confirmation of array data

We performed quantitative real-time RT-PCR to confirm the results of the whole-genome transcriptome response of *Mtb* to THZ treatment. At each of the time-points at which RNA was obtained for microarray experiments, we quantified the expression of at least one transcript. For this purpose, independent triplicate experiments were performed and RNA isolated. RT-PCR confirmed the induction of the Rv3160c, Rv3161c at 1 h; Rv3614c, Rv3615c, Rv3616c (data not shown) at 4 h and Rv2710, Rv2745c, Rv3065c at all time points in response to the 1x and 4x MIC THZ treatment. Rv0251c induced at all time points in response to 4x MIC only (Table 1). Therefore, the results obtained in our microarray experiments could be independently validated. We also tested the changes in the expression of three *Mtb* genes, σ^B^, Rv2745c and *emr*E, via RT-PCR following treatment with 0.5x MIC for different periods of time. However, at this concentration, treatment with THZ had no impact on the expression of any of these three genes (not shown).

### Damage to *Mtb* cell-envelope by THZ

**Figure 2:**
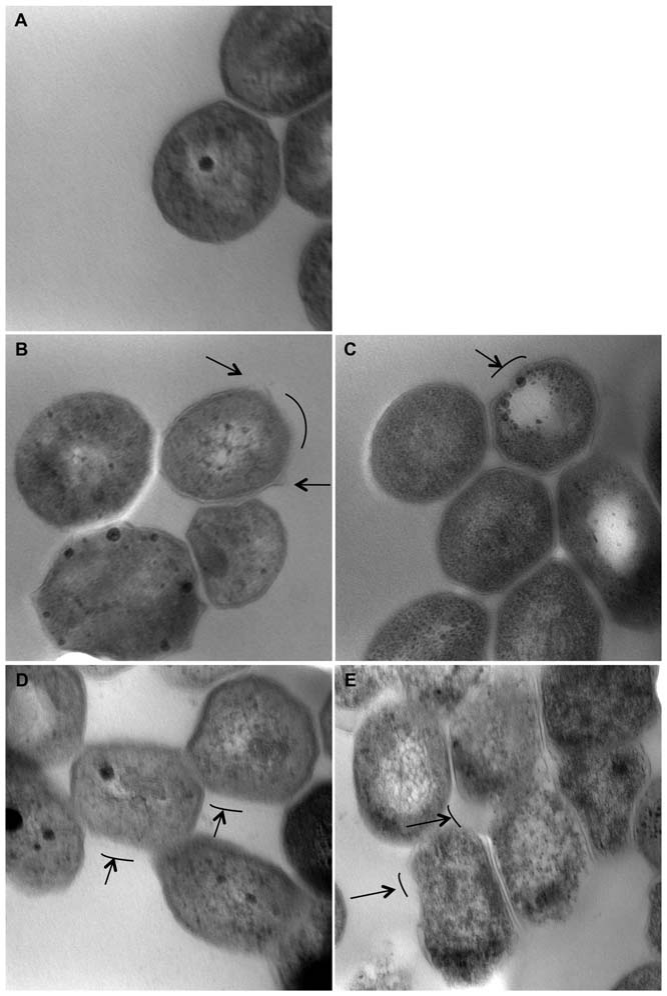
TEM images of unexposed *Mtb* CDC 1551 control (A) and following incubation for 6 hr (B, C) and 24 hr (D, E) with 1x MIC (B, D)/4x MIC (C, E) THZ respectively. The arrows and lines highlight areas of noticeable cell wall damage.

Due to the complete induction of the σ^B^ operon, and the recently documented role of this factor in protecting *Mtb* against cell-envelope damage, we hypothesized that THZ may damage or modulate *Mtb* cell-envelope. To address this question, we directly analyzed the effect of THZ treatment on *Mtb* cell-envelope by transmission electron microscopy (TEM). While *Mtb* not treated with THZ appeared to have normal envelope morphology (Figure 2A), those treated with either 1x (Figure 2B) or 4x (Figure 2C) dose of THZ for 6 hr exhibited discernable changes in the envelope morphology. A total of three fields (4–6 cells/field) from each condition were examined. An average of ∼33% (in 1x MIC, 6 h) to ∼66% (in 4x MIC, 24 h) of the examined cells showed damage. *Mtb* treated with either a 1x (Figure 2D) or 4x (Figure 2E) dose of THZ for 24 hr exhibited extensively more damage to their cell-envelope. In fact, in these samples an apparent loss of the integrity of the cell-envelope was visible. These changes to the cell-envelope morphology increased both as a function of time, as well as THZ concentration.

### Role of the σ^H^/σ^H^/σ^B^ network in protecting *Mtb* from the stress generated by THZ

**Figure 3:**
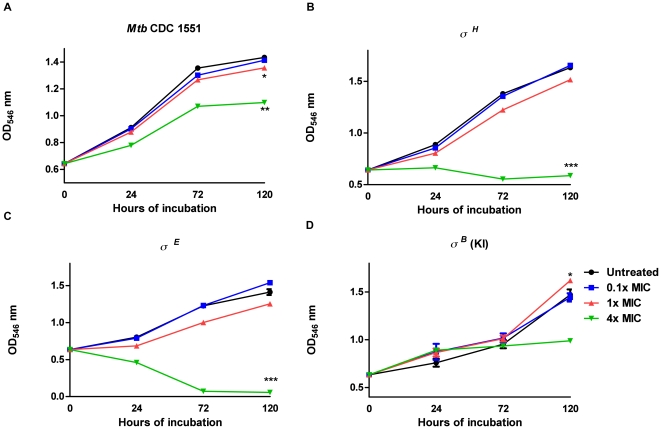
Thioridazine (THZ) inhibited the growth of *Mtb* CDC1551 (A), sigH mutant (B), *sigE* mutant (C) and σ^B^ Knock-In (KI) mutant (D) in a dose-dependent manner. *, p<0.05 **, p<0.01 ***, p<0.001 were considered to indicate 1 (0.1 x MIC), 10 (1 x MIC) and 40 (4 x MIC) µg/ml THZ-treated groups where the OD values differed significantly compared to the untreated control group. The results represent mean ± SD values from three experiments. σ^B^ over expression was induced by the addition of acetamide to 0.2%.

In *Mtb*, the alternate ECF σfactors σ^H^ and σ^E^ are intricately linked to the principal minor σfactor σ^B^ [28], [29]. σ^H^ or σ^E^ can independently induce the expression of σ^B^. In the wake of a clear induction of the σ^B^-expresson in *Mtb* by THZ, we hypothesized that a network of these σfactors is important for protecting *Mtb* from the stress caused by THZ mediated cell-envelope. We therefore studied the effect of THZ on the viability of *Mtb* and its isogenic Δ–σ^H^ and Δ–σ^E^ deletion mutants (Figure 3), as well as a strain where the expression of σ^B^ could be conditionally induced, under the control of the acetamidase promoter (*Mtb*: σ^ B^-KI). All four strains, (*Mtb*, *Mtb*:Δ–σ^ H^, *Mtb*:Δ–σ^E^ and *Mtb*: σ^ B^-KI) were either treated with 0.l x, 1x or 4x THZ or left untreated, and their viability was observed over the course of the time. The *Mtb*: Δ–σ^H^ (Figure 3B) and the *Mtb*:Δ–σ^E^ (Figure 3C) appeared to be significantly more sensitive to a high-dose of THZ, relative to *Mtb* (Figure 3A). However, the decrease in the viability of *Mtb* to a high level of THZ treatment could be rescued in the *Mtb*:σ^B^-KI strain expressing high levels of σ^B^ (Figure 3D).

### Modulation of intracellular redox potential by THZ

**Figure 4:**
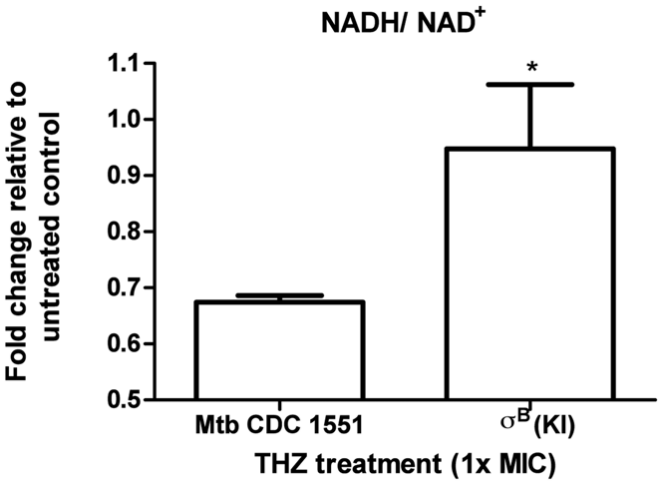
Changes in redox status of NADH/NAD^+^ during treatment of *Mtb* CDC 1551 and σ^B^ (KI) mutant with THZ. The results represent mean ± SD values from three experiments. σ^B^ over expression induced by the addition of acetamide to 0.2%.

Previous studies have shown that THZ interacts with and inhibits the activity of type-II NADH dehydrogenase, encoded by the *ndh* gene [22]. This enzyme catalyzes electron transfer from NAD(P)H to quinones, thus replenishing the NAD(P)^+^ pool, and regenerating intracellular potential. The increased expression of *ndh* by THZ treatment in *Mtb* may be an attempt by the pathogen to overcome the inhibition of this crucial enzymatic activity. We therefore studied if treatment with THZ diminished the NADH/NAD^+^ ratio. Further, since the *Mtb* σ^B^ (KI) strain appeared to protect against THZ mediated stress, we studied if the NADH/NAD^+^ ratio would be protected in this strain. We observed a significant decrease in the NADH/NAD^+^ ratio in *Mtb* following THZ treatment (Figure 4). While the decline in the intracellular redox status was reflected in a decreased NADH/NAD^+^ ratio in both the strains, there was a significant (p = 0.016) increase in this ratio in the σ^B^ (KI) strain, compared to *Mtb* CDC1551 (Figure 4). These results clearly show that induction of σ^B^ and presumably its downstream regulon rescue the declining intracellular redox potential in *Mtb* after THZ stress.

## Discussion

MDR TB has necessitated the discovery of novel compounds with activity against *Mtb* [1]. Ideally, these compounds would not only have sterilizing activity against *Mtb*, but would also shorten regimen times, and act through mechanisms different from those employed by the existing frontline and secondary anti-TB drugs. Phenothiazines such as chlorpromazine, thioridazine, promethazine and methdilazine show promising in vitro activity against clinical strains of *Mtb* regardless to their antibiotic susceptibility. While THZ has some undesirable side-effects, its potential as an antimicrobial, especially as an anti-mycobacterial can't be ignored, particularly in resource-poor countries with high MDR-TB burden. The side effects of THZ are generally very mild. While severe side-effects have been reported, these are extremely rare [10], [11], [17]. Therefore, we believe that THZ should be seriously considered as an alternative for the therapy of MDR-TB infection. Moreover, it is active against *Mtb* in a starved state and within phagocytes [15]. It also exhibits synergism with RIF and STR [5]. It targets the type II NADH dehydrogenase and succinate dehydrogenase and disrupts aerobic respiration under micro- aerobic conditions [50]. THZ significantly reduces bacterial burden in a mouse model of TB [19] and shows promising activity in a mouse model of MDR-TB.

Our results show that treatment with THZ leads to rapid and specific gene-expression responses in *Mtb*, particularly causing the repression of many genes belonging to “Lipid Metabolism” and “Information Pathways”, hence slowing down many important biosynthetic pathways. We speculate that perhaps these energetically expensive processes can't be sustained by the pathogen in the unfavorable environment due to the cell-envelope damage caused by THZ. Additional experiments are however required to address this hypothesis.

*Mtb* gene-expression response to THZ includes the putative Rv3160c/Rv3161c operon, which is also induced by Tricosan, SRI967 and SRI9190 [47]. The Rv3161c encoded dioxygenase likely encodes an aromatic dioxygenase that could degrade the halogenated diphenyl ether structure contained in these drugs. Consistent with this assessment, the Rv3160c/Rv3161c operon is not induced by treatment with SRI22, whose benzene ring is not halogenated. The expression of the Rv3065 gene, which encodes a multidrugs-transport integral membrane protein EmrE, and another putative efflux pump gene, Rv1634, was also induced following THZ treatment. THZ has been reported to act as an efflux pump inhibitor in *Mtb* [10], [11]. Our results prompt us to speculate that the efflux pump inhibition caused by THZ leads to the over-expression of the *emr*E and Rv1634 genes, as a potential mechanism by the pathogen to resist this drug. In *M. smegmatis,* deletion of the Rv3065 homolog causes increased susceptibility to a number of drugs including ethidium bromide, acriflavin and fluoroquinolones. THZ and related compounds are predicted to act via the *emr*E-encoded efflux pump [10], [11], [51]. The induction of *ndh* and efflux-pump (*emr*E) genes upon treatment of *Mtb* with THZ confirms the data presented by Boshoff et al., [22], and Amaral et al., [10], [11], respectively.

*Mtb* genes involved in other regulatory circuits, with defined roles in virulence and pathogenesis, such as the σ^B^ regulon and the Esx secretion pathway were also induced. The *Mtb* σ^H^ [28]–[30], σ^E^ [35], [52] and σ^B^ [37] operons have been very well studied. A growing body of evidence indicates that these operons are induced in response to a pleothra of *in-vitro* conditions which mimic *in-vivo* stress faced by the pathogen during the various stages of infection and disease [28]–[30], [35], [37], [41], [53]. It appears that envelope damage may serve as a key signal for *Mtb* to activate stress-defense mechanisms, and numerous *in-vitro* conditions may be able to replicate this. Damage to the envelope may inhibit specific targets, e.g. *ndh*, thus causing changes to the intracellular redox potential. Our results (Figure 4) indicate that the σ^B^ regulon protects *Mtb* from these changes. Our results, along with those of Fontan et al [37], show that *Mtb* responses to oxidative and envelope-damage stress converge at σ^B^ and Rv2745c. Further research is therefore warranted to study the role of these *Mtb* transcription factors.

The deployment of the σ^B^ regulon appears to be an attempt by the pathogen to protect against this stress. This has implications for the *in-vivo* growth of *Mtb*, where it is likely to face similar stress. The Rv2745c encoded transcription factor is a key member of the *Mtb* σ^H^/σ^E^/σ^B^ network, and its activity is induced in response to various stress conditions (Mehra et al, unpublished work from our group). It activates the expression of ATP-dependent Clp proteolysis [30], [54], [55], and may also protect the *Mtb* envelope by suppressing proton leakage [38]. The increase in the expression of the Esx genes (Table S2A) either results from the damage to the *Mtb* cell-envelope by THZ or may reflect increased secretion of virulence related substrates by the pathogen. Rv1057, which encodes a seven-bladed ß-propeller trans-membrane protein [56], [57], and which is induced in host cells during infection with *Mtb* [56]; likely due to de-repression by TrcR, was also induced by THZ. This gene is also a member of the σ^E^/σ^B^ gene-expression network and has been speculated to play a role in intraphagosomal survival and metabolism of lipid substrates. The expression of the *lip*L gene, which was induced in response to THZ, is also induced by nutritional stress [58]. Envelope damage by THZ represses metabolic biosynthesis (Figure 1). The expression of the *lip*L gene may be required to degrade triacylglycerides as *Mtb* is forced to obtain energy from alternative source [58].

This study identifies key *Mtb* gene-expression networks that are impacted upon treatment with THZ - a novel, promising anti-mycobacterial compound. Our work confirms the data presented by Boshoff et al [22]. Our work also identifies one *Mtb* regulatory network (σ^B^) involved in countering its effects, enhancing our current understanding of the role played by this regulatory switch. Our work lays the foundation of future interrogation of these networks as potential drug-targets.

## Supporting Information

Table S1 Primers used for the qRT-PCR.
(0.16 MB PPT)

Table S2 Genes differentially regulated after treatment with Thioridazine at 1XMIC/4XMIC at different time points
(0.31 MB PPT)

Table S3 Induction of the *Mtb* σ^B^ regulon by THZ. (A) σ^B^ is up regulated in THZ-induced *Mtb*. Further, 89 genes (out of top 100*) that are very strongly co-expressed with σ^B^ are also induced by this treatment. (p<0.0001). *The gene expression correlation values were computed as pair-wise Pearson correlations using the microarray data sets from the Boshoff et al 2004 [22] study and Dr. Schoolnik's lab (unpublished data) and are imported from TBDB. “Data was obtained from the NIAID BioHealthBase BRC online through the web site at http://www.biohealthbase.org. (B) Comparison between genes under σ^B^ regulation during SDS stress (Fontan et al 2009) [37] and THZ stress [this study]: 57 genes are common among 73 (p<0.0001). (C) Comparison between genes under σ^B^ regulation during diamide stress (Fontan et al 2009) [37] and THZ stress [this study]: 30 genes are common among 41(p<0.004). (D) Comparison between genes under σ^B^ regulation during VAN [38] and THZ treatment. 32 genes out of the 37 that were induced by VAN in Mtb [38] were also induced by THZ [this study]. Genes from the 100 member σ^B^- regulon that were commonly regulated by THZ and the other listed treatments (SDS, Diamide, VAN) are shown in bold. The genes not shown in bold indicate oppositely regulated genes between THZ and the other listed treatments.
(0.29 MB DOC)

Figure S1 A two-dimensional clustering heat map shows *Mtb* genes that were expressed in a statistically significant manner in at least one post-THZ treatment time-point (1, 2, 4, 6 hr) in either the 1x or the 4x treatment experiment. The values are expressed in base 2 logarithmic scale. The intensity of red color correlates with a higher degree of expression in *Mtb* treated with THZ relative to the control Mtb strain, while the intensity of the blue color correlates with a lower degree of expression in *Mtb* treated with THZ relative to the control *Mtb* strain. The genes with the highest degree of blue and red color corresponds to a log2 fold change value of 5.81 (i.e. a numeric fold change of >56 fold).
(0.16 MB PPT)

Figure S2 Venn diagrams. Venn diagrams show the degree of association between the transcriptional induction of *Mtb* genes in response to 1x (A) or 4x (B) THZ treatment at different time points. Numbers at the intersection of more than one oval indicate the number of genes overlapping between those time points.
(0.11 MB PPT)
