# Supplementary material for: A Mycobacterium tuberculosis Sigma Factor Network Responds to Cell-Envelope Damage by the Promising Anti-Mycobacterial Thioridazine
Source: PLoS One. 2010 Apr 8;5(4):e10069. doi: 10.1371/journal.pone.0010069 (PMC2851646; doi:10.1371/journal.pone.0010069)
Supplement: Table S1 — Primers used for the qRT-PCR. (0.16 MB PPT) [file pone.0010069.s001.ppt]

## Slide 1
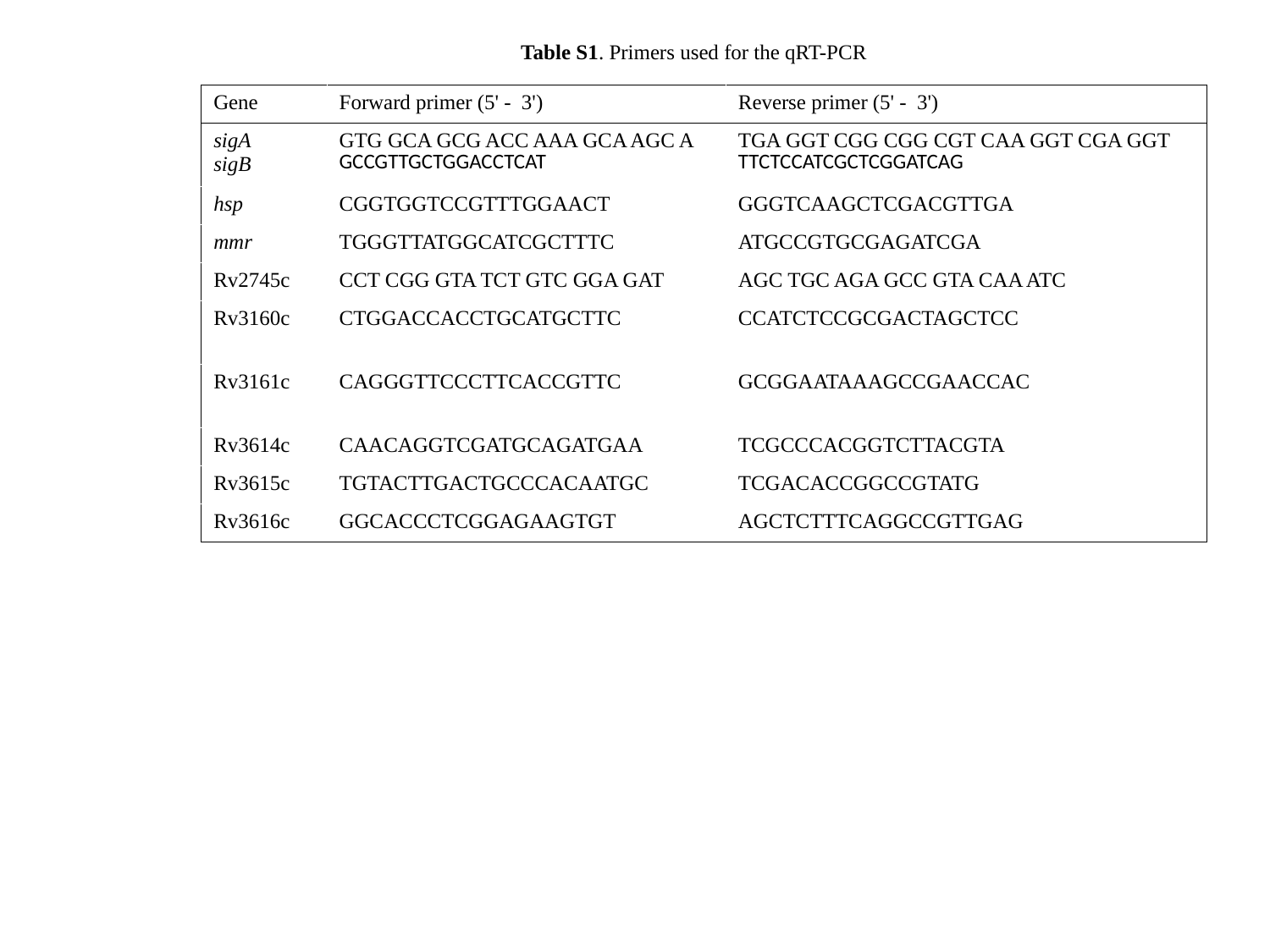

Table S1. Primers used for the qRT-PCR
| Gene | Forward primer (5' - 3') | Reverse primer (5' - 3') |
| --- | --- | --- |
| sigA sigB | GTG GCA GCG ACC AAA GCA AGC A GCCGTTGCTGGACCTCAT | TGA GGT CGG CGG CGT CAA GGT CGA GGT TTCTCCATCGCTCGGATCAG |
| hsp | CGGTGGTCCGTTTGGAACT | GGGTCAAGCTCGACGTTGA |
| mmr | TGGGTTATGGCATCGCTTTC | ATGCCGTGCGAGATCGA |
| Rv2745c | CCT CGG GTA TCT GTC GGA GAT | AGC TGC AGA GCC GTA CAA ATC |
| Rv3160c | CTGGACCACCTGCATGCTTC | CCATCTCCGCGACTAGCTCC |
| Rv3161c | CAGGGTTCCCTTCACCGTTC | GCGGAATAAAGCCGAACCAC |
| Rv3614c | CAACAGGTCGATGCAGATGAA | TCGCCCACGGTCTTACGTA |
| Rv3615c | TGTACTTGACTGCCCACAATGC | TCGACACCGGCCGTATG |
| Rv3616c | GGCACCCTCGGAGAAGTGT | AGCTCTTTCAGGCCGTTGAG |
