# Supplementary material for: A Mycobacterium tuberculosis Sigma Factor Network Responds to Cell-Envelope Damage by the Promising Anti-Mycobacterial Thioridazine
Source: PLoS One. 2010 Apr 8;5(4):e10069. doi: 10.1371/journal.pone.0010069 (PMC2851646; doi:10.1371/journal.pone.0010069)
Supplement: Table S2 — Genes differentially regulated after treatment with Thioridazine at 1XMIC/4XMIC at different time points (0.31 MB PPT) [file pone.0010069.s002.ppt]

## Slide 1
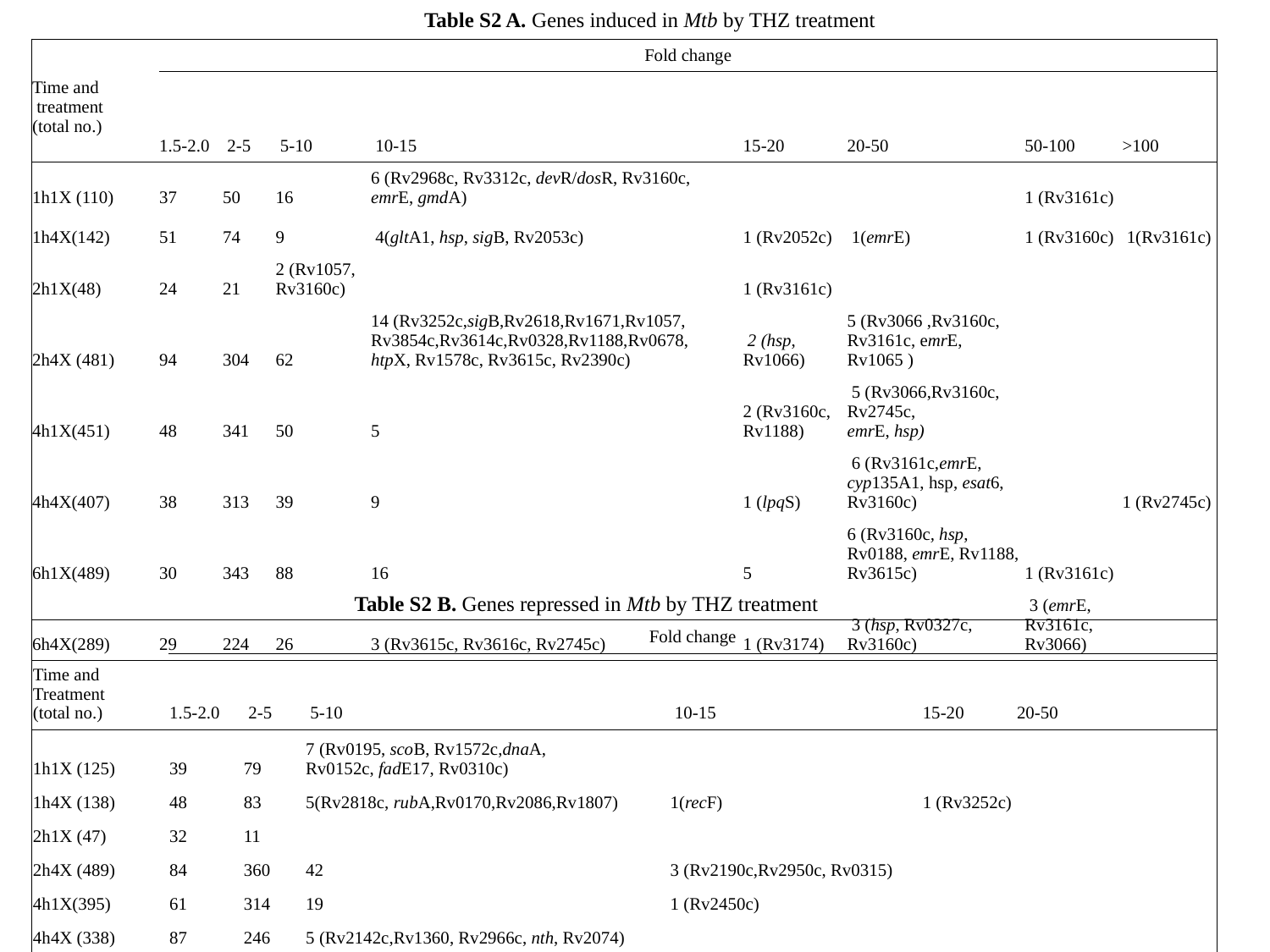

Table S2 A. Genes induced in Mtb by THZ treatment
| Time and treatment (total no.) | Fold change | | | | | | | |
| --- | --- | --- | --- | --- | --- | --- | --- | --- |
| | 1.5-2.0 | 2-5 | 5-10 | 10-15 | 15-20 | 20-50 | 50-100 | >100 |
| 1h1X (110) | 37 | 50 | 16 | 6 (Rv2968c, Rv3312c, devR/dosR, Rv3160c, emrE, gmdA) | | | 1 (Rv3161c) | |
| 1h4X(142) | 51 | 74 | 9 | 4(gltA1, hsp, sigB, Rv2053c) | 1 (Rv2052c) | 1(emrE) | 1 (Rv3160c) | 1(Rv3161c) |
| 2h1X(48) | 24 | 21 | 2 (Rv1057, Rv3160c) | | 1 (Rv3161c) | | | |
| 2h4X (481) | 94 | 304 | 62 | 14 (Rv3252c,sigB,Rv2618,Rv1671,Rv1057, Rv3854c,Rv3614c,Rv0328,Rv1188,Rv0678, htpX, Rv1578c, Rv3615c, Rv2390c) | 2 (hsp, Rv1066) | 5 (Rv3066 ,Rv3160c, Rv3161c, emrE, Rv1065 ) | | |
| 4h1X(451) | 48 | 341 | 50 | 5 | 2 (Rv3160c, Rv1188) | 5 (Rv3066,Rv3160c, Rv2745c, emrE, hsp) | | |
| 4h4X(407) | 38 | 313 | 39 | 9 | 1 (lpqS) | 6 (Rv3161c,emrE, cyp135A1, hsp, esat6, Rv3160c) | | 1 (Rv2745c) |
| 6h1X(489) | 30 | 343 | 88 | 16 | 5 | 6 (Rv3160c, hsp, Rv0188, emrE, Rv1188, Rv3615c) | 1 (Rv3161c) | |
| 6h4X(289) | 29 | 224 | 26 | 3 (Rv3615c, Rv3616c, Rv2745c) | 1 (Rv3174) | 3 (hsp, Rv0327c, Rv3160c) | 3 (emrE, Rv3161c, Rv3066) | |
Table S2 B. Genes repressed in Mtb by THZ treatment
| Time and Treatment (total no.) | Fold change | | | | | |
| --- | --- | --- | --- | --- | --- | --- |
| | 1.5-2.0 | 2-5 | 5-10 | 10-15 | 15-20 | 20-50 |
| 1h1X (125) | 39 | 79 | 7 (Rv0195, scoB, Rv1572c,dnaA, Rv0152c, fadE17, Rv0310c) | | | |
| 1h4X (138) | 48 | 83 | 5(Rv2818c, rubA,Rv0170,Rv2086,Rv1807) | 1(recF) | 1 (Rv3252c) | |
| 2h1X (47) | 32 | 11 | | | | |
| 2h4X (489) | 84 | 360 | 42 | 3 (Rv2190c,Rv2950c, Rv0315) | | |
| 4h1X(395) | 61 | 314 | 19 | 1 (Rv2450c) | | |
| 4h4X (338) | 87 | 246 | 5 (Rv2142c,Rv1360, Rv2966c, nth, Rv2074) | | | |
| 6h1X (411) | 58 | 325 | 27 | | | 1 (Rv2450c) |
| 6h4X(218) | 78 | 135 | 5 (Rv2074, Rv0696,desA1, Rv3130c, Rv2030c) | | | |
