# Supplementary material for: A Mycobacterium tuberculosis Sigma Factor Network Responds to Cell-Envelope Damage by the Promising Anti-Mycobacterial Thioridazine
Source: PLoS One. 2010 Apr 8;5(4):e10069. doi: 10.1371/journal.pone.0010069 (PMC2851646; doi:10.1371/journal.pone.0010069)
Supplement: Table S3 — Induction of the Mtb σB regulon by THZ. (A) σB is up regulated in THZ-induced Mtb. Further, 89 genes (out of top 100*) that are very strongly co-expressed with σB are also induced by this treatment. (p<0.0001). *The gene expression correlation values were computed as pair-wise Pearson correlations using the microarray data sets from the Boshoff et al 2004 [22] study and Dr. Schoolnik's lab (unpublished data) and are imported from TBDB. “Data was obtained from the NIAID BioHealthBase BRC online through the web site at http://www.biohealthbase.org. (B) Comparison between genes under σB regulation during SDS stress (Fontan et al 2009) [37] and THZ stress [this study]: 57 genes are common among 73 (p<0.0001). (C) Comparison between genes under σB regulation during diamide stress (Fontan et al 2009) [37] and THZ stress [this study]: 30 genes are common among 41(p<0.004). (D) Comparison between genes under σB regulation during VAN [38] and THZ treatment. 32 genes out of the 37 that were induced by VAN in Mtb [38] were also induced by THZ [this study]. Genes from the 100 member σB- regulon that were commonly regulated by THZ and the other listed treatments (SDS, Diamide, VAN) are shown in bold. The genes not shown in bold indicate oppositely regulated genes between THZ and the other listed treatments. (0.29 MB DOC) [file pone.0010069.s003.doc]

|  |  |  | |  |  |  |  |  |  |  |  |  |  |  |  |
| --- | --- | --- | --- | --- | --- | --- | --- | --- | --- | --- | --- | --- | --- | --- | --- |
| **Table S3**. Induction of the *Mtb* σ^B^ regulon by THZ. | | | | | | | | | | | | | | | |
| **A** | | | | | **B** | | | | **C** | | | | **D** | | |
| **Rank** | **Rv#** | | **Gene** |  | **Sl. No.** | **Rv#** | **Gene** |  | **Sl. No.** | **Rv#** | **Gene** |  | **Sl. No.** | **Rv#** | **Gene** |
| **1** | **Rv 2744c** | | ***35kd_ag*** |  | 1 | ORF01418 |  |  | **1** | **ORF01178** |  |  | **1** | **Rv0140** |  |
| **2** | **Rv 0251c** | | ***hsp*** |  | **2** | **ORF04174** |  |  | **2** | **ORF04548** |  |  | **2** | **Rv0188** |  |
| **3** | **Rv2711** | | ***ide*R** |  | **3** | **ORF04343** |  |  | **3** | **Rv0250c** |  |  | **3** | **Rv0251c** | ***hsp*** |
| **4** | **Rv2745c** | |  |  | **4** | **ORF04372** |  |  | **4** | **Rv0251c** | [***hsp***](http://genome-www4.stanford.edu/cgi-bin/SMD/source/sourceResult?choice=Gene&option=Name&criteria=hsp) |  | **4** | **Rv0549** |  |
| **5** | **Rv2694c** | |  |  | **5** | **ORF05088** |  |  | 5 | Rv0325 |  |  | **5** | **Rv0677c** | ***mmp*S5** |
| **6** | **Rv3206c** | | ***moe*B1** |  | **6** | **ORF08402** |  |  | **6** | **Rv0331** |  |  | 6 | Rv0750 |  |
| **7** | **Rv0188** | |  |  | **7** | **ORF08416** |  |  | 7 | Rv0332 |  |  | **7** | **Rv0834c** | ***PE_PGRS14*** |
| **8** | **Rv2053c** | | ***fxs*A** |  | 8 | Rv0005 | *gyr*B |  | **8** | **Rv0350** | [***dna*K**](http://genome-www4.stanford.edu/cgi-bin/SMD/source/sourceResult?choice=Gene&option=Name&criteria=dnaK) |  | **8** | **Rv1072** |  |
| **9** | **Rv2497c** | | ***pdh*A** |  | **9** | **Rv0064** |  |  | **9** | **Rv0384c** | [***clp*B**](http://genome-www4.stanford.edu/cgi-bin/SMD/source/sourceResult?choice=Gene&option=Name&criteria=clpB) |  | **9** | **Rv1073** |  |
| 10 | Rv 2688c | |  |  | 10 | Rv0106 |  |  | **10** | **Rv0426c** |  |  | **10** | **Rv1221** | ***sig*E** |
| **11** | **Rv 0678** | |  |  | **11** | **Rv0116c** |  |  | **11** | **Rv0678** |  |  | **11** | **Rv1350** | ***fab*G** |
| **12** | **Rv 1222** | |  |  | 12 | Rv0158 |  |  | **12** | **Rv0846c** |  |  | **12** | **Rv1404** |  |
| **13** | **Rv 1460** | |  |  | **13** | **Rv0188** |  |  | **13** | **Rv0847** | [***lpq*S**](http://genome-www4.stanford.edu/cgi-bin/SMD/source/sourceResult?choice=Gene&option=Name&criteria=lpqS) |  | 13 | Rv1955 |  |
| **14** | **Rv 0384c** | | ***clp*B** |  | 14 | Rv0196 |  |  | 14 | Rv1049 |  |  | **14** | **Rv1991c** |  |
| **15** | **Rv 0898c** | |  |  | 15 | Rv0232 |  |  | **15** | **Rv1050** |  |  | **15** | **Rv2035** |  |
| **16** | **Rv 0563** | | ***htp*X** |  | **16** | **Rv0340** |  |  | 16 | Rv1129c |  |  | **16** | **Rv2050** |  |
| **17** | **Rv 2500c** | | ***fad*E19** |  | **17** | **Rv0348** |  |  | **17** | **Rv1130** |  |  | **17** | **Rv2052c** |  |
| **18** | **Rv1894c** | |  |  | **18** | **Rv0464c** |  |  | 18 | Rv1131 | [*glt*A1](http://genome-www4.stanford.edu/cgi-bin/SMD/source/sourceResult?choice=Gene&option=Name&criteria=gltA1) |  | **18** | **Rv2053c** | ***fxs*A** |
| **19** | **Rv2111c** | |  |  | **19** | **Rv0465c** |  |  | **19** | **Rv1265** |  |  | **19** | **Rv2115c** |  |
| **20** | **Rv1072** | |  |  | **20** | **Rv0549c** |  |  | **20** | **Rv1285** | [***cys*D**](http://genome-www4.stanford.edu/cgi-bin/SMD/source/sourceResult?choice=Gene&option=Name&criteria=cysD) |  | **20** | **Rv2466c** |  |
| **21** | **Rv0064** | |  |  | **21** | **Rv0550c** |  |  | **21** | **Rv1286** | [***cys*N**](http://genome-www4.stanford.edu/cgi-bin/SMD/source/sourceResult?choice=Gene&option=Name&criteria=cysN) |  | **21** | **Rv2497c** | ***pdh*A** |
| **22** | **Rv2115c** | |  |  | **22** | **Rv0586** |  |  | **22** | **Rv1460** |  |  | **22** | **Rv2517c** |  |
| **23** | **Rv0140** | |  |  | **23** | **Rv0677c** | ***mmp*S5** |  | **23** | **Rv1461** |  |  | **23** | **Rv2602** |  |
| **24** | **Rv2816c** | |  |  | **24** | **Rv0679c** |  |  | **24** | **Rv1462** |  |  | **24** | **Rv2659c** |  |
| **25** | **Rv0724** | | ***spp*A** |  | **25** | **Rv0758** | ***pho*R** |  | **25** | **Rv1464** | [***csd***](http://genome-www4.stanford.edu/cgi-bin/SMD/source/sourceResult?choice=Gene&option=Name&criteria=Rv1464) |  | **25** | **Rv2694c** |  |
| **26** | **Rv0834c** | | ***PE_PGRS14*** |  | **26** | **Rv0857** |  |  | **26** | **Rv1465** |  |  | **26** | **Rv2711** | ***ide*R** |
| **27** | **Rv3287c** | | ***rsb*W** |  | **27** | **Rv0872c** | ***PE_PGRS*** |  | **27** | **Rv1466** |  |  | **27** | **Rv2745c** |  |
| **28** | **Rv3161c** | |  |  | **28** | **Rv1174c** |  |  | 28 | Rv1652 | [*arg*C](http://genome-www4.stanford.edu/cgi-bin/SMD/source/sourceResult?choice=Gene&option=Name&criteria=argC) |  | **28** | **Rv3066** |  |
| **29** | **Rv 2743c** | |  |  | 29 | Rv1216c |  |  | 29 | Rv1653 | [*arg*J](http://genome-www4.stanford.edu/cgi-bin/SMD/source/sourceResult?choice=Gene&option=Name&criteria=argJ) |  | **29** | **Rv3188** |  |
| 30 | Rv0810c | |  |  | **30** | **Rv1218c** |  |  | 30 | Rv1654 | [*arg*B](http://genome-www4.stanford.edu/cgi-bin/SMD/source/sourceResult?choice=Gene&option=Name&criteria=argB) |  | **30** | **Rv3287c** | ***rsb*W** |
| **31** | **Rv3173c** | |  |  | **31** | **Rv1219c** |  |  | **31** | **Rv1766** |  |  | **31** | **Rv3288c** | ***usf*Y** |
| **32** | **Rv2052c** | |  |  | **32** | **Rv1285** | ***cys*D** |  | 32 | Rv1767 |  |  | **32** | **Rv3290c** | ***lat*** |
| **33** | **Rv2829c** | |  |  | **33** | **Rv1332** |  |  | **33** | **Rv1909c** | [***fur*A**](http://genome-www4.stanford.edu/cgi-bin/SMD/source/sourceResult?choice=Gene&option=Name&criteria=furA) |  | 33 | Rv3334 |  |
| **34** | **Rv2602** | |  |  | **34** | **Rv1356c** |  |  | **34** | **Rv1992c** | [***ctp*G**](http://genome-www4.stanford.edu/cgi-bin/SMD/source/sourceResult?choice=Gene&option=Name&criteria=ctpG) |  | **34** | **Rv3503c** | ***fdx*D** |
| **35** | **Rv1991c** | |  |  | 35 | Rv1364c | *rsb*U |  | **35** | **Rv1993c** |  |  | **35** | **Rv3742c** |  |
| **36** | **Rv3408** | |  |  | **36** | **Rv1497** | ***lip*L** |  | **36** | **Rv2016** |  |  | 36 | Rv3854c |  |
| **37** | **Rv2498c** | | ***cit*E** |  | 37 | Rv1574 |  |  | 37 | Rv2242 |  |  | 37 | Rv3917c |  |
| **38** | **Rv2526** | |  |  | **38** | **Rv1670** |  |  | **38** | **Rv2640c** |  |  |  |  |  |
| **39** | **Rv2374c** | | ***hrc*A** |  | **39** | **Rv1671** |  |  | **39** | **Rv2745c** |  |  |  |  |  |
| **40** | **Rv2050** | |  |  | **40** | **Rv1831** |  |  | **40** | **Rv2780** | [***ald***](http://genome-www4.stanford.edu/cgi-bin/SMD/source/sourceResult?choice=Gene&option=Name&criteria=ald) |  |  |  |  |
| **41** | **Rv3503c** | | ***fdx*D** |  | **41** | **Rv1832** | ***gcv*B** |  | 41 | Rv3833 |  |  |  |  |  |
| 42 | Rv3610c | | *fts*H |  | 42 | Rv1960c |  |  |  |  |  |  |  |  |  |
| **43** | **Rv3052c** | | ***nrd*I** |  | **43** | **Rv2016** |  |  |  |  |  |  |  |  |  |
| **44** | **Rv3288c** | | ***usf*Y** |  | **44** | **Rv2091c** |  |  |  |  |  |  |  |  |  |
| **45** | **Rv3197** | |  |  | 45 | Rv2110c | *prc*B |  |  |  |  |  |  |  |  |
| **46** | **Rv1846c** | |  |  | **46** | **Rv2111c** |  |  |  |  |  |  |  |  |  |
| **47** | **Rv3526** | |  |  | **47** | **Rv2115c** |  |  |  |  |  |  |  |  |  |
| **48** | **Rv1909c** | | ***fur*A** |  | 48 | Rv2182c |  |  |  |  |  |  |  |  |  |
| **49** | **Rv1221** | | ***sig*E** |  | **49** | **Rv2204c** |  |  |  |  |  |  |  |  |  |
| **50** | **Rv3290c** | | ***lat*** |  | 50 | Rv2255c |  |  |  |  |  |  |  |  |  |
| **51** | **Rv3064c** | |  |  | **51** | **Rv2256c** |  |  |  |  |  |  |  |  |  |
| **52** | **Rv2496c** | | ***pdh*B** |  | 52 | Rv2555c | *ala*S |  |  |  |  |  |  |  |  |
| **53** | **Rv3596c** | | ***clp*C1** |  | **53** | **Rv2660c** |  |  |  |  |  |  |  |  |  |
| **54** | **Rv0677c** | | ***mmp*S5** |  | **54** | **Rv2688c** |  |  |  |  |  |  |  |  |  |
| 55 | Rv3917c | | *par*B |  | **55** | **Rv2711** | ***ide*R** |  |  |  |  |  |  |  |  |
| **56** | **Rv2517c** | |  |  | **56** | **Rv2712c** |  |  |  |  |  |  |  |  |  |
| **57** | **Rv0274** | |  |  | 57 | Rv2755c | *hsd*S*'* |  |  |  |  |  |  |  |  |
| **58** | **Rv0874c** | |  |  | **58** | **Rv2816c** |  |  |  |  |  |  |  |  |  |
| **59** | **Rv0549** | |  |  | **59** | **Rv2865** |  |  |  |  |  |  |  |  |  |
| **60** | **Rv1073** | |  |  | **60** | **Rv2869c** | ***rip*** |  |  |  |  |  |  |  |  |
| **61** | **Rv3334** | |  |  | **61** | **Rv2989** |  |  |  |  |  |  |  |  |  |
| **62** | **Rv3188** | |  |  | **62** | **Rv3180c** |  |  |  |  |  |  |  |  |  |
| **63** | **Rv2495** | | ***pdhC*** |  | **63** | **Rv3288c** |  |  |  |  |  |  |  |  |  |
| **64** | **Rv3527** | |  |  | 64 | Rv3452 |  |  |  |  |  |  |  |  |  |
| 65 | Rv2077c | |  |  | **65** | **Rv3610c** | ***fts*H** |  |  |  |  |  |  |  |  |
| **66** | **Rv1586c** | |  |  | **66** | **Rv3642c** |  |  |  |  |  |  |  |  |  |
| **67** | **Rv1056** | |  |  | **67** | **Rv3765c** |  |  |  |  |  |  |  |  |  |
| **68** | **Rv2925c** | |  |  | **68** | **Rv3766** |  |  |  |  |  |  |  |  |  |
| **69** | **Rv0758** | | ***phoR*** |  | **69** | **Rv3767c** |  |  |  |  |  |  |  |  |  |
| **70** | **Rv2659c** | |  |  | **70** | **Rv3802c** |  |  |  |  |  |  |  |  |  |
| 71 | Rv1955 | |  |  | **71** | **Rv3810** | ***pir*G** |  |  |  |  |  |  |  |  |
| 72 | Rv2516c | |  |  | **72** | **Rv3825c** | ***pks*2** |  |  |  |  |  |  |  |  |
| 73 | Rv0983 | | *pepD* |  | 73 | Rv3903c |  |  |  |  |  |  |  |  |  |
| **74** | **Rv2583c** | | ***relA*** |  |  |  |  |  |  |  |  |  |  |  |  |
| **75** | **Rv2527** | |  |  |  |  |  |  |  |  |  |  |  |  |  |
| 76 | Rv3854c | | *ethA* |  |  |  |  |  |  |  |  |  |  |  |  |
| **77** | **Rv0088** | |  |  |  |  |  |  |  |  |  |  |  |  |  |
| **78** | **Rv1350** | | ***fabG*** |  |  |  |  |  |  |  |  |  |  |  |  |
| 79 | Rv0750 | |  |  |  |  |  |  |  |  |  |  |  |  |  |
| **80** | **Rv2035** | |  |  |  |  |  |  |  |  |  |  |  |  |  |
| **81** | **Rv2466c** | |  |  |  |  |  |  |  |  |  |  |  |  |  |
| **82** | **Rv2036** | |  |  |  |  |  |  |  |  |  |  |  |  |  |
| **83** | **Rv0786c** | |  |  |  |  |  |  |  |  |  |  |  |  |  |
| **84** | **Rv3182** | |  |  |  |  |  |  |  |  |  |  |  |  |  |
| **85** | **Rv2372c** | |  |  |  |  |  |  |  |  |  |  |  |  |  |
| **86** | **Rv3802c** | |  |  |  |  |  |  |  |  |  |  |  |  |  |
| 87 | Rv2664 | |  |  |  |  |  |  |  |  |  |  |  |  |  |
| **88** | **Rv0426c** | |  |  |  |  |  |  |  |  |  |  |  |  |  |
| **89** | **Rv1584c** | |  |  |  |  |  |  |  |  |  |  |  |  |  |
| **90** | **Rv3238c** | |  |  |  |  |  |  |  |  |  |  |  |  |  |
| **91** | **Rv3066** | |  |  |  |  |  |  |  |  |  |  |  |  |  |
| **92** | **Rv1404** | |  |  |  |  |  |  |  |  |  |  |  |  |  |
| **93** | **Rv0412c** | |  |  |  |  |  |  |  |  |  |  |  |  |  |
| **94** | **Rv2841c** | | ***nus*A** |  |  |  |  |  |  |  |  |  |  |  |  |
| **95** | **Rv1992c** | | ***ctp*G** |  |  |  |  |  |  |  |  |  |  |  |  |
| **96** | **Rv0560c** | |  |  |  |  |  |  |  |  |  |  |  |  |  |
| **97** | **Rv3742c** | |  |  |  |  |  |  |  |  |  |  |  |  |  |
| **98** | **Rv0747** | | ***PE_PGRS10*** |  |  |  |  |  |  |  |  |  |  |  |  |
| **99** | **Rv2828c** | |  |  |  |  |  |  |  |  |  |  |  |  |  |
| **100** | **Rv3053c** | | ***nrd*H** |  |  |  |  |  |  |  |  |  |  |  |  |
