# Supplementary figures and images for: A Mycobacterium tuberculosis Sigma Factor Network Responds to Cell-Envelope Damage by the Promising Anti-Mycobacterial Thioridazine
Source: PLoS One. 2010 Apr 8;5(4):e10069. doi: 10.1371/journal.pone.0010069 (PMC2851646; doi:10.1371/journal.pone.0010069)

## Slide 1
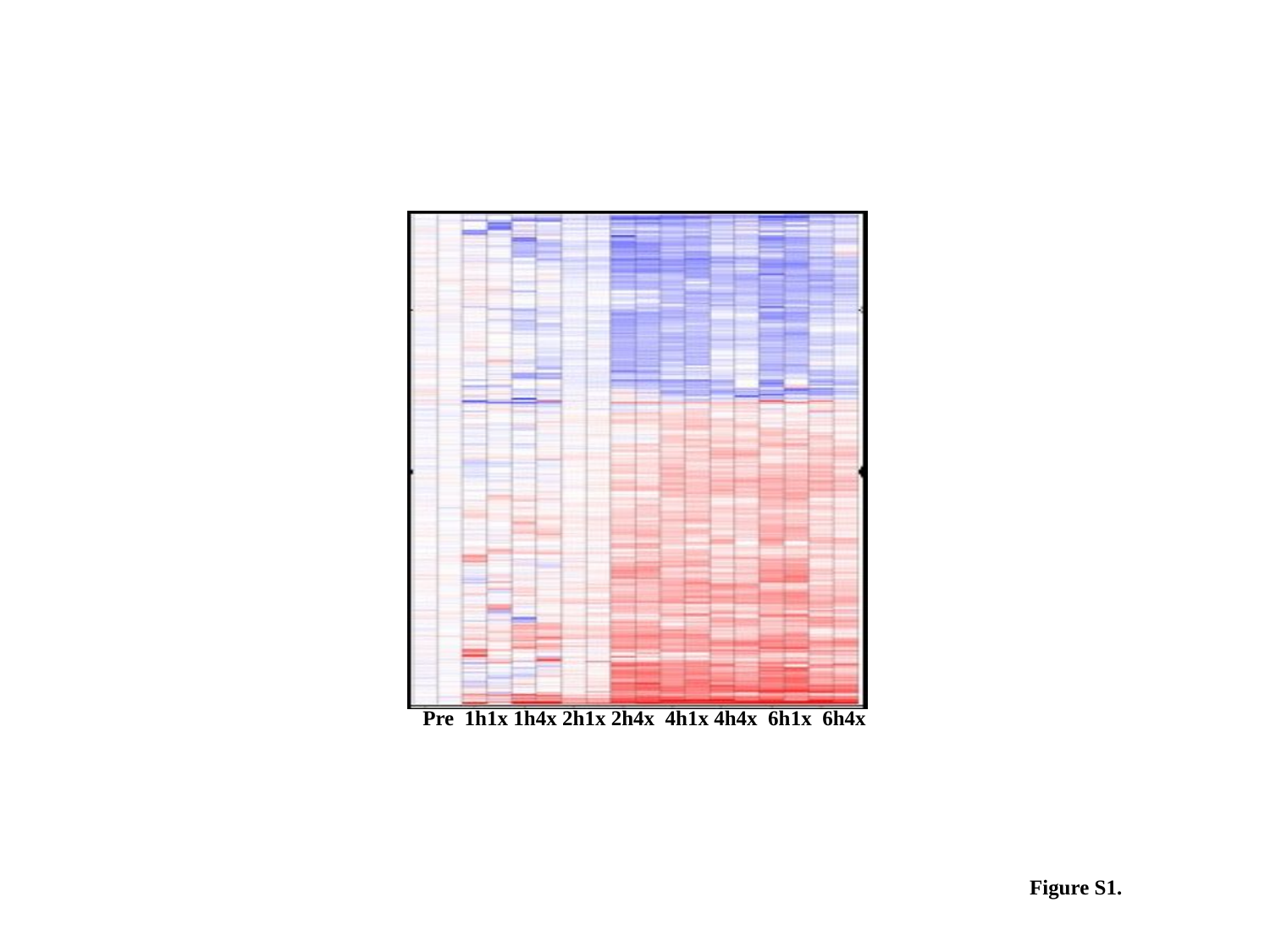

Pre 1h1x 1h4x 2h1x 2h4x 4h1x 4h4x 6h1x 6h4x
Figure S1.

Supplement: Figure S1 — A two-dimensional clustering heat map shows Mtb genes that were expressed in a statistically significant manner in at least one post-THZ treatment time-point (1, 2, 4, 6 hr) in either the 1x or the 4x treatment experiment. The values are expressed in base 2 logarithmic scale. The intensity of red color correlates with a higher degree of expression in Mtb treated with THZ relative to the control Mtb strain, while the intensity of the blue color correlates with a lower degree of expression in Mtb treated with THZ relative to the control Mtb strain. The genes with the highest degree of blue and red color corresponds to a log2 fold change value of 5.81 (i.e. a numeric fold change of >56 fold). (0.16 MB PPT) [file pone.0010069.s004.ppt]

## Slide 1
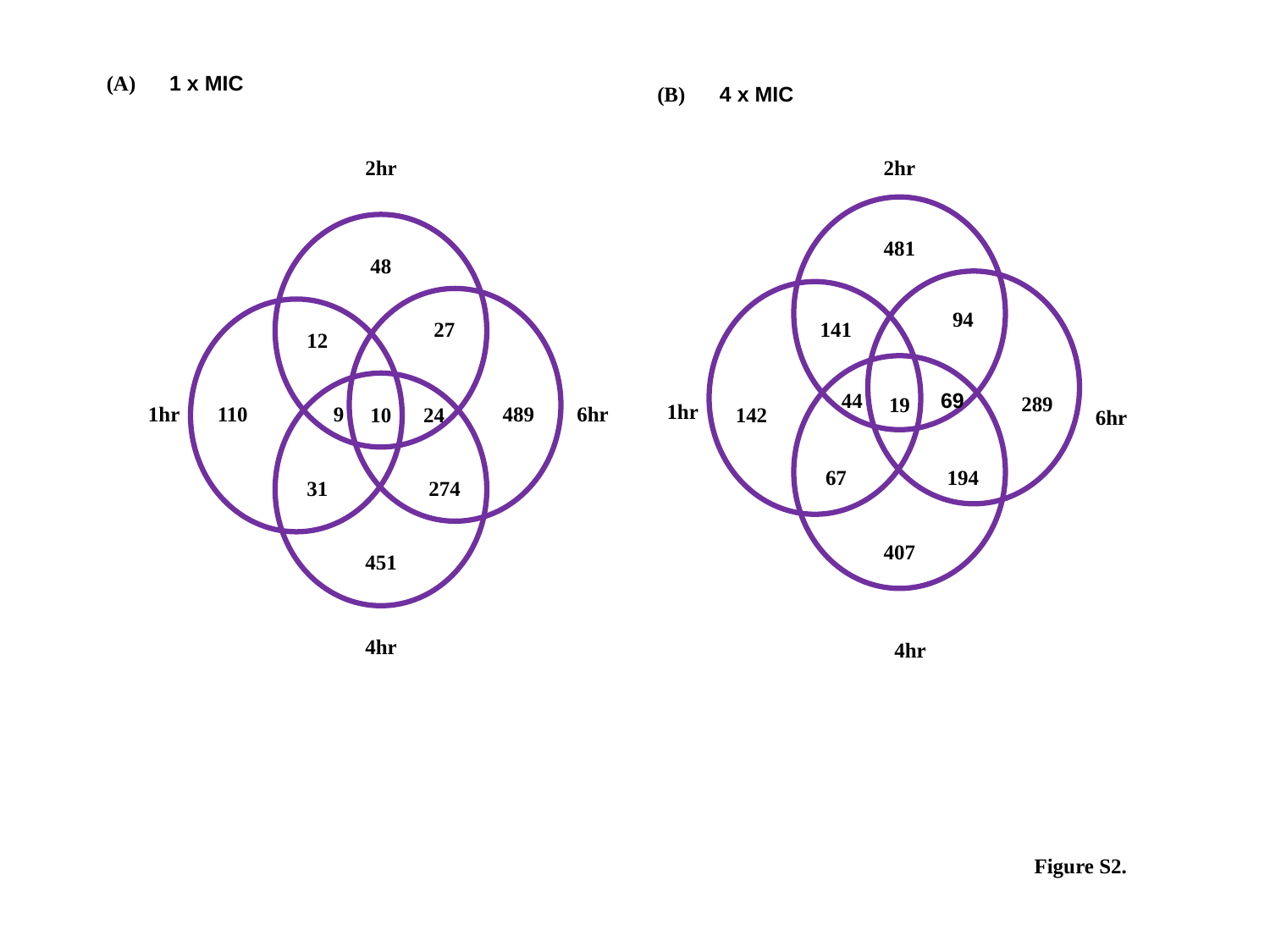

(a)
(A)
1 x MIC
(B)
4 x MIC
2hr
2hr
481
48
94
27
141
12
44
69
289
19
1hr
1hr
110
9
489
6hr
10
24
142
6hr
67
194
31
274
407
451
4hr
4hr
Figure S2.

Supplement: Figure S2 — Venn diagrams. Venn diagrams show the degree of association between the transcriptional induction of Mtb genes in response to 1x (A) or 4x (B) THZ treatment at different time points. Numbers at the intersection of more than one oval indicate the number of genes overlapping between those time points. (0.11 MB PPT) [file pone.0010069.s005.ppt]
